# Supplementary material for: Discovery of synthetic lethal interactions from large-scale pan-cancer perturbation screens
Source: Nat Commun. 2022 Dec 14;13:7748. doi: 10.1038/s41467-022-35378-z (PMC9751287; doi:10.1038/s41467-022-35378-z)
Supplement: Supplementary file 1 — Supplementary Information [file 41467_2022_35378_MOESM1_ESM.pdf]

Supplementary Information

Supplementary notes

Supplementary Note 1. Consolidating experimentally identified SL interactions from CRISPR screens

To compare SLIdR's predictions with established SL interactions, we focused on experimentally identified SL interactions from *in vitro* SL screens and combinatorial CRISPR screens. While the list of interactions for the former was obtained from Lee *et al.'s*[1] study, for CRISPR-based SL interactions, we consolidated 24,651 experimentally identified SL interactions from 10 combinatorial CRISPR screens[2–11] with 13 shared interactions across the screens. Since SLIdR predicts only SL interactions, we retained pairs with negative genetic interactions (GI) scores from the works of Horlbeck *et al.*[4] and Norman *et al.*[7]. DeWeirdt *et al.*[11] provided a ranked list of SL partners for *PARP1, BCL2L1, and MCL1*, and we retained the first 100 for each. It should be noted that the majority of these experiments were performed in a few selective cell lines, and several studies focused only on identifying paralog pairs and SL partners for specific driver genes.

Supplementary figures and figure legends

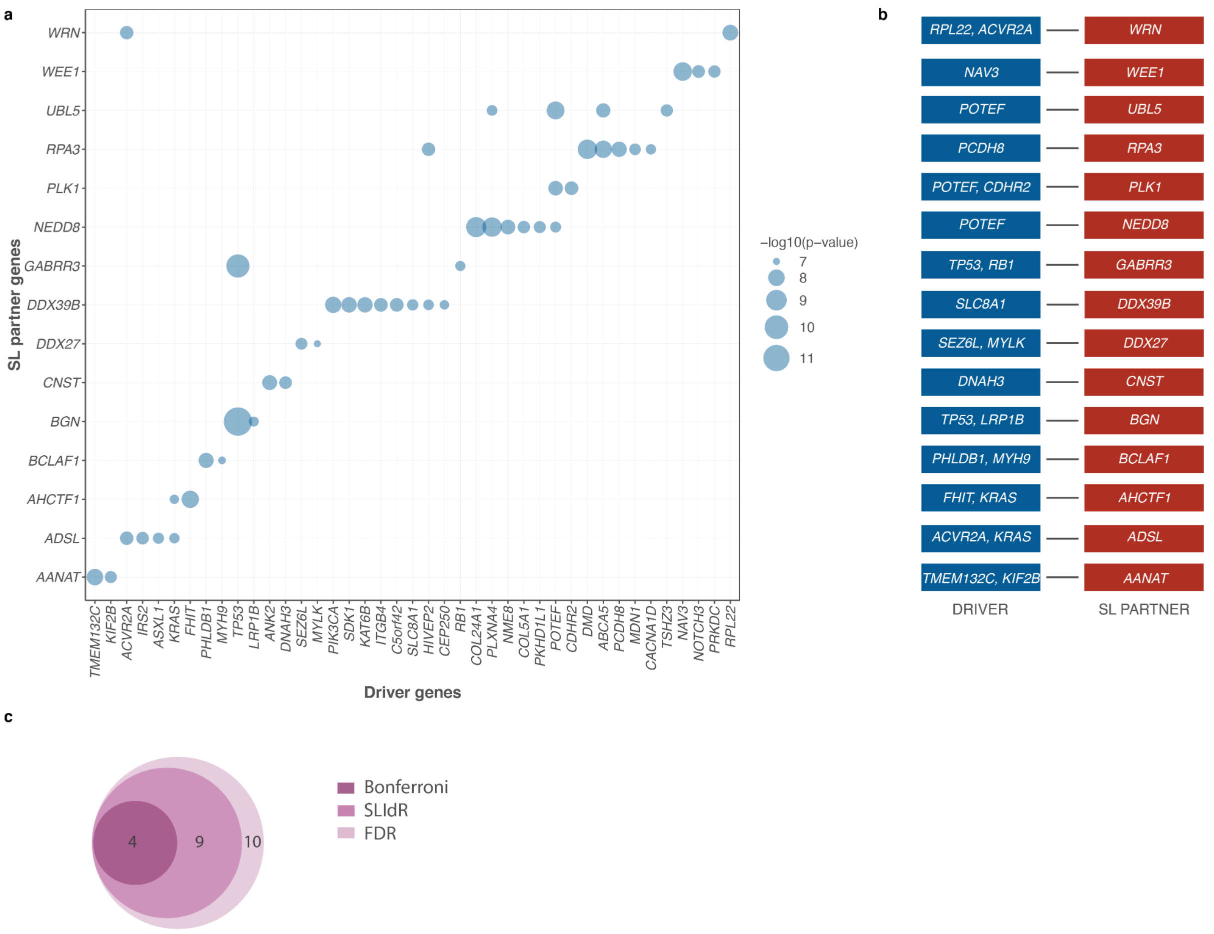

Supplementary Fig. 1. Confounding in pan-cancer analysis and comparison of results from different multiple testing methods. a Bubble-plot summarizing the significance (-log10(p-value)) of different driver genes (x-axis) pairing with the same SL partner gene (y-axis) as predicted by SLIdR in the pan-cancer analysis after filtering out false positives from multiple testing. The p-values are computed using one-sided IH-test. **b** Corresponding list of significant SL pairs after accounting for confounding mutations and performing causal inference using matching-based potential outcome models. **c** An example illustrating the consensus between SLIdR hits and hits resulting from controlling the false discovery rate (FDR) at 10%.

Venn-diagram comparing SLIdR hits and hits reported after multiple testing corrections with FDR (10%) and Bonferroni (10%) methods in liver cancer.

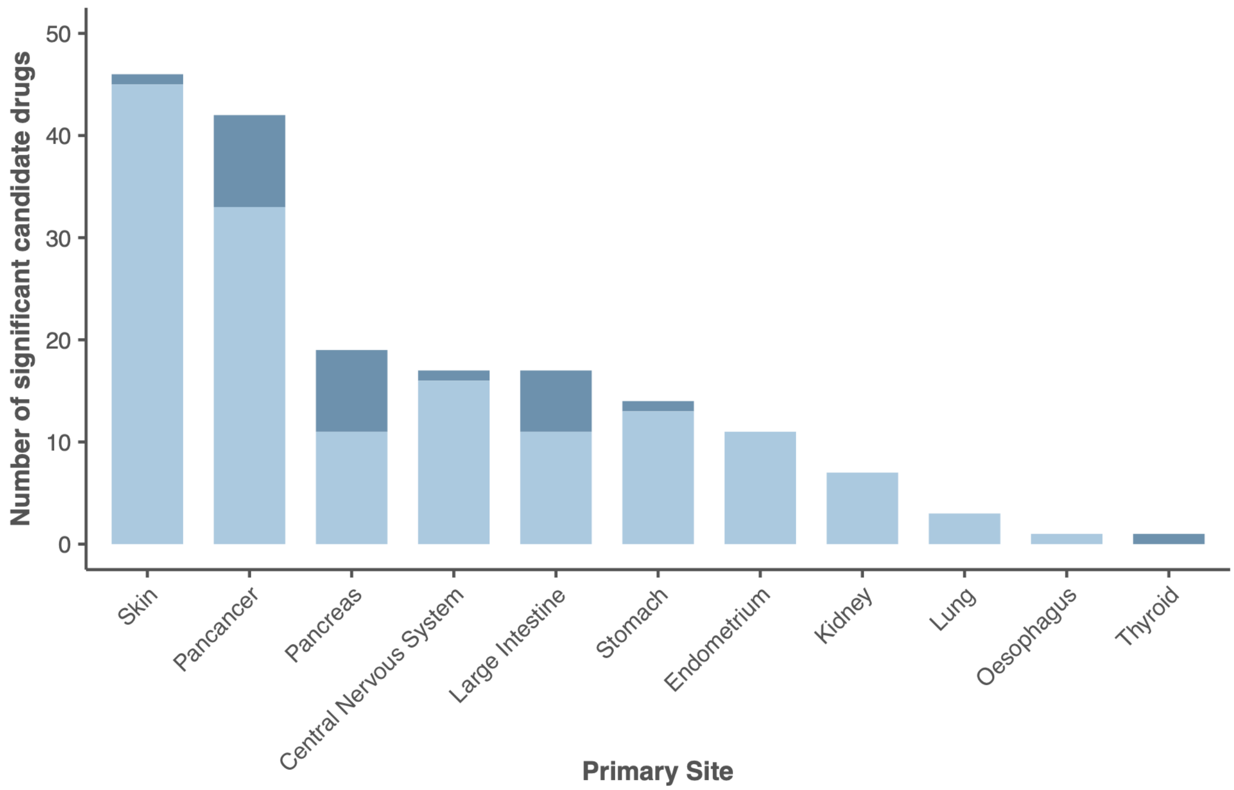

Supplementary Fig. 2. Significant drugs from PRISM screen. Barplot of the number of significant candidate drugs (α = 0.1) in pan-cancer and cancer type-specific settings on the primary PRISM repurposing dataset. The dark blue stacks correspond to drugs that are significant after multiple testing correction (q-values $<= 0.2$).

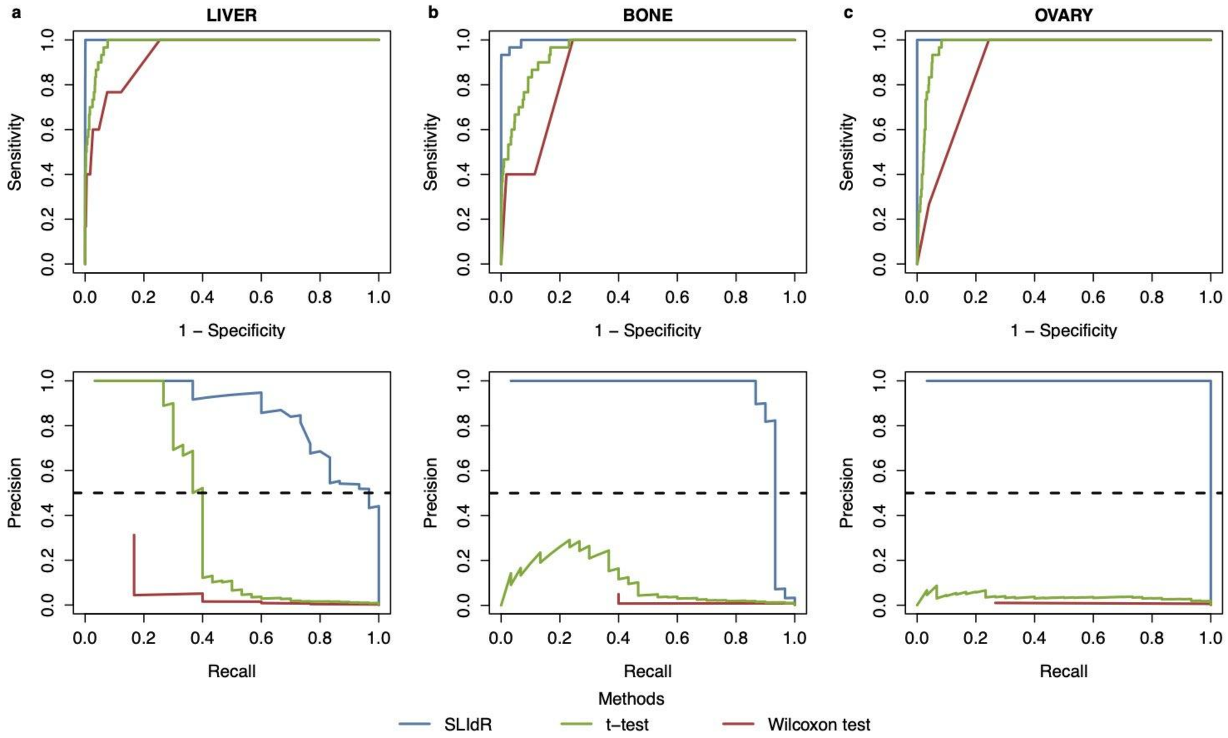

Supplementary Fig. 3. Performance assessment on simulated data. ROC curves (top) and Precision-Recall curves (bottom) for predictions of SL pairs using SLIdR, Wilcoxon-test, and t-test on simulated data for **a** Liver, **b** Bone, and **c** Ovary site-specific cancers, respectively. The simulated data for liver, bone, and ovarian cancers included 13, 7, and 14 cell lines, respectively. The mutation data were taken from the cancer type-specific analysis on Project DRIVE dataset. For a given cancer type, the corresponding viabilities were simulated such that the data comprised 30 ground truth SL pairs between driver genes and perturbed genes.

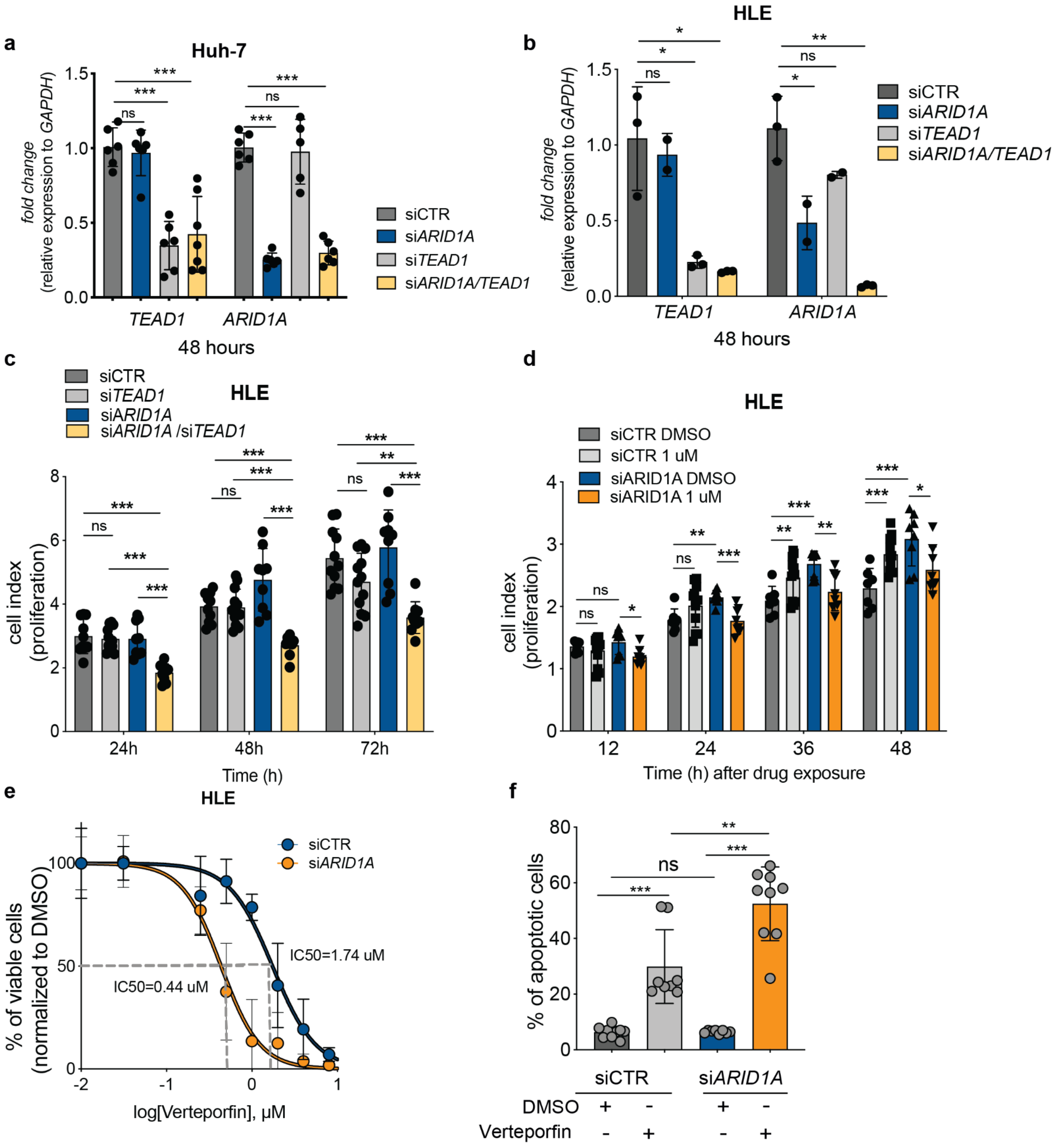

Supplementary Fig. 4. Functional validation of SL interaction between *ARID1A* and *TEAD1*. **a**,**b** RNA expression level (fold-change) of *ARID1A* and *TEAD1* relative to *GAPDH* in (**a**) Huh-7 and (**b**) HLE. RNA levels were assessed by quantitative real-time PCR (qPCR). **c**,**d** Cell proliferation assay in HLE cell line (**c**) transfected with control siRNA or *ARID1A* and *TEAD1* siRNA alone or in combination, or (**d**) transfected with control siRNA or *ARID1A* siRNA

and treated with DMSO or verteporfin (1uM). **e** Dose-response curve of verteporfin in HLE cells transfected with control or *ARID1A* siRNA. **f** Apoptosis assay using AnnexinV and propidium iodide (PI) staining of HLE cells transfected with control or ARID1A siRNA 48 hours after treatment with DMSO or verteporfin (0.5uM). Error bars represent mean (+/- SD) from n≥2 replicated. For all experiments performed, statistical significance was assessed by two-sided multiple t-tests (* P < 0.05, ** P < 0.01, *** P < 0.001).

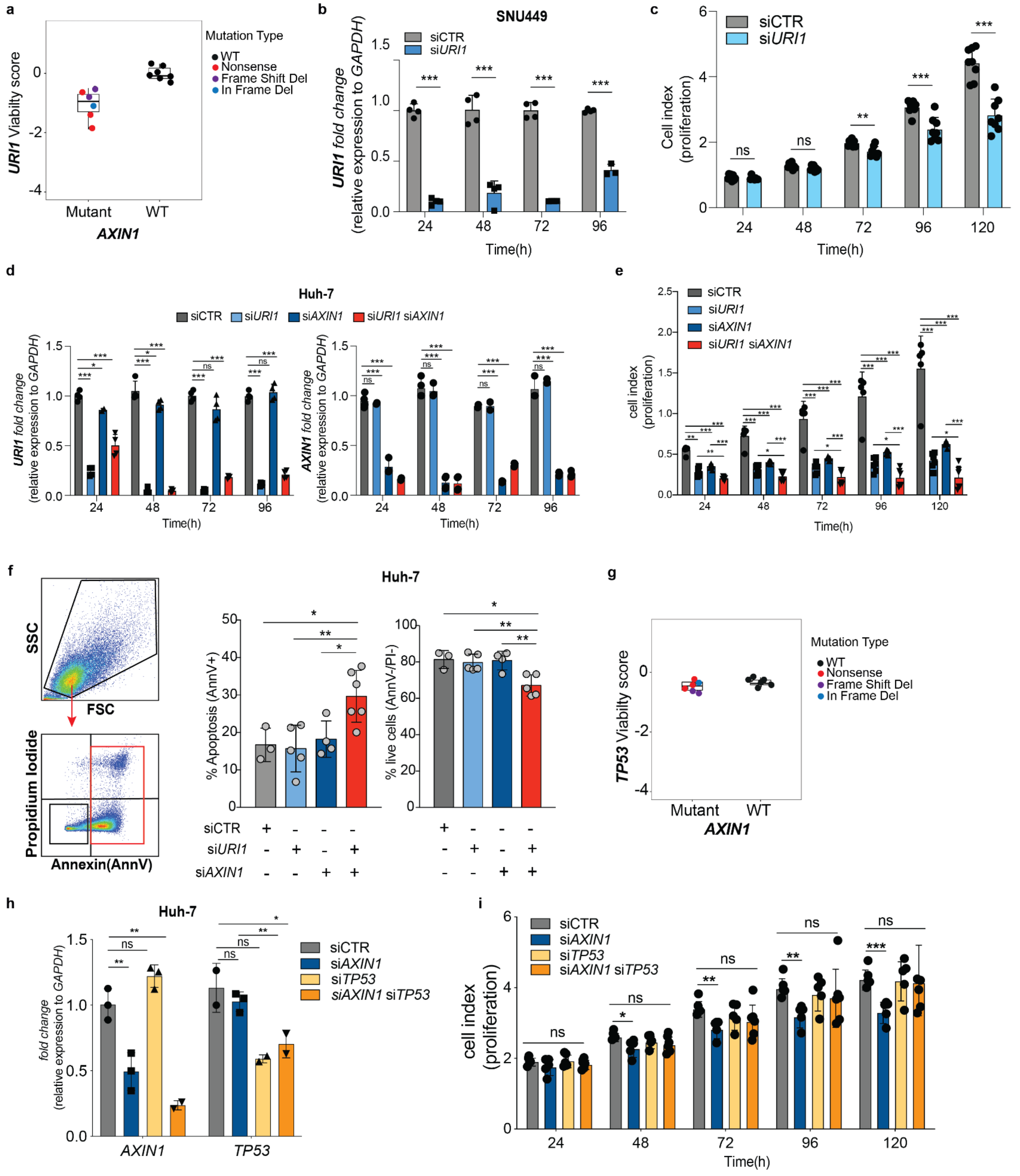

Supplementary Fig. 5. Functional validation of SL interaction between *AXIN1* and *URI1* and non-lethal interaction between *AXIN1* and *TP53* as negative control. a Viability scores of *AXIN1* mutant vs wild-type (WT) HCC cell lines with *URI1* knockdown from Project DRIVE

dataset, where n = 13 HCC cell lines subject to *URI1* knockdown experiment. Data are presented as boxplots: Mutant = {min = -1.894, lower (1st Qu.) = -1.2975, middle (median) = -0.9515, upper (3rd Qu.) = -0.70675, max = -0.495} and WT = {min = -0.285, lower (1st Qu.) = -0.1760, middle (median) = -0.0870, upper (3rd Qu.) = 0.16950, max = 0.323}. **b** RNA expression level (fold-change) of *URI1* relative to *GAPDH* in SNU449 cells transfected with control siRNA (grey) or *URI1* siRNA (blue). RNA levels were assessed by quantitative real-time PCR (qPCR). **c** Cell proliferation assay in SNU449 cell line (*AXIN1* mutated) transfected with control siRNA (grey) or *URI1* siRNA (blue). **d** RNA expression levels (fold-change) of *URI1* (left) and *AXIN1* (right) relative to *GAPDH* in Huh-7 cell line transfected with control siRNA (grey), *URI1* siRNA (light blue), *AXIN1* siRNA (dark blue) or both (red). **e** Cell proliferation assay in Huh-7 cell line (*AXIN1* WT) transfected with control siRNA (grey), *URI1* siRNA (light blue), *AXIN1* siRNA (dark blue) or both (red). **f** Apoptosis assay using AnnexinV and propidium iodide (PI) staining in Huh-7 cell line (*AXIN1* wild-type) transfected with control siRNA (grey), *URI1* siRNA (light blue), *AXIN1* siRNA (dark blue) or both (red). Quantification of the mean percentage of apoptotic cells (AnnexinV+) and live cells (PI-/AnnexinV-) across the different groups (n=4) (right). **g** Viability scores of *AXIN1* mutant vs wild-type (WT) HCC cell lines with *TP53* knockdown from Project DRIVE dataset, where n = 13 HCC cell lines subject to *TP53* knockdown experiment. Data are presented as boxplots: Mutant = {min = -0.67, lower (1st Qu.) = -0.6035, middle (median) = -0.4595, upper (3rd Qu.) = -0.3260, max = -0.304} and WT = {min = -0.45, lower (1st Qu.) = -0.4175, middle (median) = -0.3670, upper (3rd Qu.) = -0.2655, max = -0.170}. **h** RNA expression levels (fold-change) of *AXIN1* and *TP53* relative to *GAPDH* in Huh-7 cell line transfected with control siRNA (grey), *TP53* siRNA (yellow), *AXIN1* siRNA (dark blue) or both (orange). RNA levels were assessed by qPCR at 72 hours post siRNA transfection. Statistics were performed assuming similar scatter. **i** Cell proliferation assay in Huh-7 cell line (*AXIN1* WT) transfected with control siRNA (grey), *TP53* siRNA (yellow), *AXIN1* siRNA (dark blue) or both (orange). Error bars represent mean (+/- SD) from n≥2 replicated. For all

experiments performed, statistical significance was assessed by two-sided multiple t-tests (* P < 0.05, ** P < 0.01, *** P < 0.001).

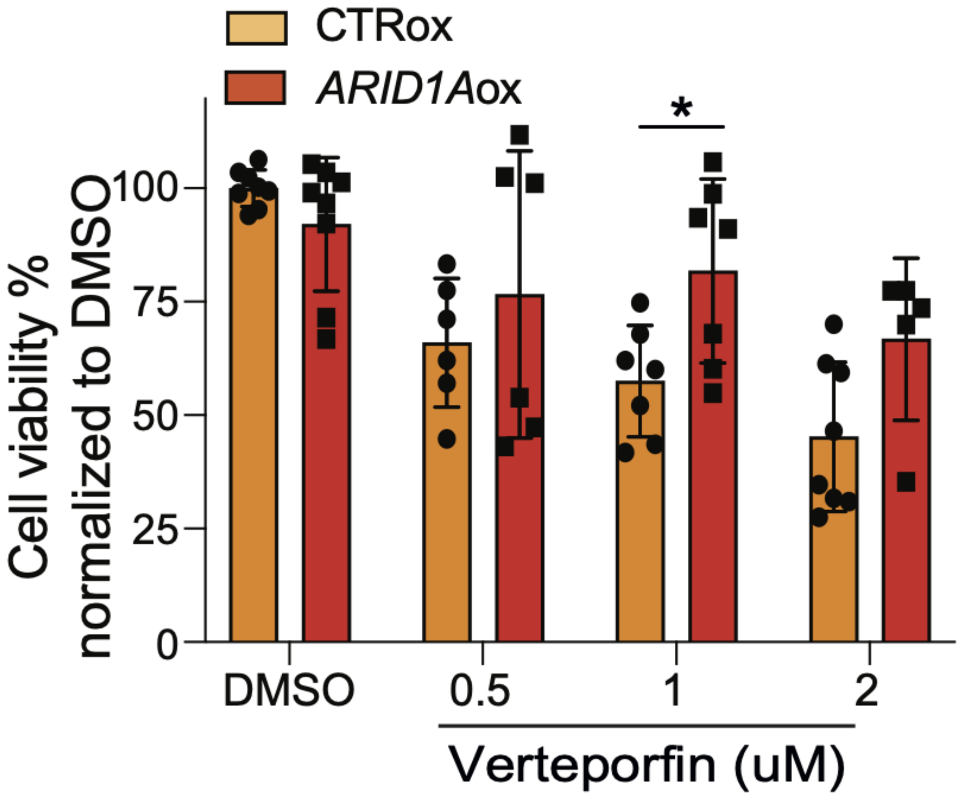

Supplementary Fig. 6. Inhibition of *TEAD1* is deleterious in *ARID1A* mutant liver cancer cells. SNU449 cells overexpressing *ARID1A* and control cells treated with vehicle (DMSO) or different dosage of verteporfin. Error bars represent mean (+/- SD) from n≥3 replicated. For all experiments performed, statistical significance was assessed by 2-way ANOVA (* P < 0.05).

References:

1. Lee, J. S. *et al.* Harnessing synthetic lethality to predict the response to cancer treatment. *Nat. Commun.* **9**, 2546 (2018).
2. Najm, F. J. *et al.* Orthologous CRISPR–Cas9 enzymes for combinatorial genetic screens. *Nature Biotechnology* **36**, 179–189 (2018).
3. Han, K. *et al.* Synergistic drug combinations for cancer identified in a CRISPR screen for pairwise genetic interactions. *Nature Biotechnology* **35**, 463–474 (2017).
4. Horlbeck, M. A. *et al.* Mapping the Genetic Landscape of Human Cells. *Cell* **174**, 953–967.e22 (2018).
5. Shen, J. P. *et al.* Combinatorial CRISPR–Cas9 screens for de novo mapping of genetic interactions. *Nature Methods* **14**, 573–576 (2017).
6. Thompson, N. A. *et al.* Combinatorial CRISPR screen identifies fitness effects of gene paralogues. *Nat. Commun.* **12**, 1302 (2021).
7. Norman, T. M. *et al.* Exploring genetic interaction manifolds constructed from rich single-cell phenotypes. *Science* **365**, 786–793 (2019).
8. Zhao, D. *et al.* Combinatorial CRISPR-Cas9 Metabolic Screens Reveal Critical Redox Control Points Dependent on the KEAP1-NRF2 Regulatory Axis. *Mol. Cell* **69**, 699–708.e7 (2018).
9. Dede, M., McLaughlin, M., Kim, E. & Hart, T. Multiplex enCas12a screens detect functional buffering among paralogs otherwise masked in monogenic Cas9 knockout screens. *Genome Biol.* **21**, 262 (2020).
10. Zhao, Y. *et al.* A one-step tRNA-CRISPR system for genome-wide genetic interaction mapping in mammalian cells. *Sci. Rep.* **9**, 14499 (2019).
11. DeWeirdt, P. C. *et al.* Genetic screens in isogenic mammalian cell lines without single cell cloning. *Nat. Commun.* **11**, 752 (2020).
