# Supplementary material for: Discovery of synthetic lethal interactions from large-scale pan-cancer perturbation screens
Source: Nat Commun. 2022 Dec 14;13:7748. doi: 10.1038/s41467-022-35378-z (PMC9751287; doi:10.1038/s41467-022-35378-z)
Supplement: Supplementary file 3 — Reporting Summary [file 41467_2022_35378_MOESM3_ESM.pdf]

nature portfolio

Corresponding author(s): Prof. Niko Beerenwinkel
Dr. Salvatore Piscuoglio

Last updated by author(s): Nov 10 2022

Reporting Summary

Nature Portfolio wishes to improve the reproducibility of the work that we publish. This form provides structure for consistency and transparency in reporting. For further information on Nature Portfolio policies, see our Editorial Policies and the Editorial Policy Checklist.

Statistics

For all statistical analyses, confirm that the following items are present in the figure legend, table legend, main text, or Methods section.

n/a	Confirmed	
☐	☒	The exact sample size (n) for each experimental group/condition, given as a discrete number and unit of measurement
☐	☒	A statement on whether measurements were taken from distinct samples or whether the same sample was measured repeatedly
☐	☒	The statistical test(s) used AND whether they are one- or two-sided<br>*Only common tests should be described solely by name; describe more complex techniques in the Methods section.*
☐	☒	A description of all covariates tested
☐	☒	A description of any assumptions or corrections, such as tests of normality and adjustment for multiple comparisons
☐	☒	A full description of the statistical parameters including central tendency (e.g. means) or other basic estimates (e.g. regression coefficient) AND variation (e.g. standard deviation) or associated estimates of uncertainty (e.g. confidence intervals)
☐	☒	For null hypothesis testing, the test statistic (e.g. F, t, r) with confidence intervals, effect sizes, degrees of freedom and P value noted<br>*Give P values as exact values whenever suitable.*
☒	☐	For Bayesian analysis, information on the choice of priors and Markov chain Monte Carlo settings
☒	☐	For hierarchical and complex designs, identification of the appropriate level for tests and full reporting of outcomes
☒	☐	Estimates of effect sizes (e.g. Cohen's d, Pearson's r), indicating how they were calculated

Our web collection on statistics for biologists contains articles on many of the points above.

Software and code

Policy information about availability of computer code

Data collection	The datasets used for the statistical analyses and predictions are publicly available and required no additional software for data collection. In the validation experiments, cell proliferation was assayed using the xCELLigence system (RTCA, ACEA Biosciences, San Diego, CA, USA), and flow cytometry was performed using BD FACSCanto II cytometer.
Data analysis	The latest version of SLIdR package is available at https://github.com/cbg-ethz/slidr along with the scripts used to process and generate the results for the paper. Other R packages used, include, Matching (v 4.9-3), tableone (v 0.9.3), dplyr (v 0.7.6), ggplot2 (v 3.2.0.9), cowplot (v 0.9.2), circlize (v 0.4.5). For the validation experiments the following softwares were used - BD, FACSDiva and FlowJo (10.5.3), Prism for MacOS v8.2.1

For manuscripts utilizing custom algorithms or software that are central to the research but not yet described in published literature, software must be made available to editors and reviewers. We strongly encourage code deposition in a community repository (e.g. GitHub). See the Nature Portfolio guidelines for submitting code & software for further information.

Data

Policy information about availability of data

All manuscripts must include a data availability statement. This statement should provide the following information, where applicable:

- Accession codes, unique identifiers, or web links for publicly available datasets
- A description of any restrictions on data availability
- For clinical datasets or third party data, please ensure that the statement adheres to our policy

The raw shRNA data has already been published as a part of Project DRIVE (https://data.mendeley.com/datasets/y3ds55n88r/4) and all the mutation and copy number data from CCLE is available at https://portals.broadinstitute.org/ccle. The MutSig 2CV v3.1 MAF file for each cancer type is available at http://firebrowse.org/. The processed project DRIVE data for running SLIdR in pan-cancer and cancer type-specific settings are available at https://doi.org/10.6084/m9.figshare.21508065.v4. The PRISM drug-response dataset and the CRISPR dataset (Project Achilles 20Q2) are available at https://depmap.org/portal/download/. All these datasets are publicly available. The validation experimental data generated in this study are provided in the Source data file.

Human research participants

Policy information about studies involving human research participants and Sex and Gender in Research.

Reporting on sex and gender	n/a
Population characteristics	n/a
Recruitment	n/a
Ethics oversight	n/a

Note that full information on the approval of the study protocol must also be provided in the manuscript.

Field-specific reporting

Please select the one below that is the best fit for your research. If you are not sure, read the appropriate sections before making your selection.

☒ Life sciences ☐ Behavioural & social sciences ☐ Ecological, evolutionary & environmental sciences

For a reference copy of the document with all sections, see nature.com/documents/nr-reporting-summary-flat.pdf

Life sciences study design

All studies must disclose on these points even when the disclosure is negative.

Sample size	No statistical method was used to predetermine sample size.
Data exclusions	In our experiments, some of the data points from technical replicates were removed due to likely cell plating and drug dosage anomalies of the multichannel pipette, or were removed as they were impossible values that could result from errors in the execution of the experiments or data entry.
Replication	All the experiments were performed at least three times and reported in the manuscript only when reproducible.
Randomization	For the in vivo experiments, CAMs were allocated randomly to each condition.
Blinding	For the in vivo model, CAMs were screened for tumour formation blindly by two independent scientists.

Reporting for specific materials, systems and methods

We require information from authors about some types of materials, experimental systems and methods used in many studies. Here, indicate whether each material, system or method listed is relevant to your study. If you are not sure if a list item applies to your research, read the appropriate section before selecting a response.

Materials & experimental systems

n/a	Involved in the study
	[x] Antibodies
	[x] Eukaryotic cell lines
[x]	Palaeontology and archaeology
	[x] Animals and other organisms
[x]	Clinical data
[x]	Dual use research of concern

Methods

n/a	Involved in the study
[x]	ChIP-seq
	[x] Flow cytometry
[x]	MRI-based neuroimaging

Antibodies

Antibodies used	anti-PARP (cell signalling #9542, 1:1000); anti-Beta Actin (Sigma #A5441, 1:2000); LI-COR Biosciences 926-68070 (Goat anti-mouse IgG, 1:10000); LI-COR Biosciences 926-68070 (Goat Anti-Rabbit IgG Antibody; 1:10000)
Validation	Antibodies have been previously validated by the manufacturer.<br><br>LI-COR Biosciences antibodies were tested by Dot Blot and/or solid-phase adsorbed for minimal cross-reactivity with human, goat, rat, and horse serum proteins, but may cross-react with immunoglobulins from other species. The conjugate has been specifically tested and qualified for Western blot and In-Cell Western applications.<br><br>PARP Antibody detects endogenous levels of full length PARP1 (116 kDa), as well as the large fragment (89 kDa) of PARP1 resulting from caspase cleavage. The antibody does not cross-react with related proteins or other PARP isoforms. Polyclonal antibodies are produced by immunizing animals with a synthetic peptide corresponding to the caspase cleavage site in PARP. Antibodies are purified by protein A and peptide affinity chromatography.<br><br>Anti-β-Actin : Monoclonal Anti-β-Actin (mouse IgG1 isotype) is derived from the AC-15 hybridoma produced by the fusion of mouse myeloma cells and splenocytes from an immunized mouse. Actin is one of the most conserved eukaryotic proteins, it is expressed in mammals and birds as at least six isoforms. Four of them represent the differentiation markers of muscle tissues and two are found practically in all cells. There are three α-actins (α-skeletal, α-cardiac, and α-smooth muscle), one β-actin (β-nonmuscle), and two γ-actins (γ-smooth muscle and γ-non-muscle). Actin isoforms show >90% overall sequence homology, but only 50–60% homology in their 18 NH2-terminal residues. The NH2-terminal region of actin appears to be a major antigenic region and may be involved in the interaction of actin with other proteins such as myosin. The antibody can be used for staining of acetone-fixed frozen sections, EM preparations, and microinjection experiments. B5, ethanol, methacam, or Bouin's solutions can be used as fixatives. The epitope recognized by the antibody is resistant to formalin-fixed and paraffin-embedding.

Eukaryotic cell lines

Policy information about cell lines and Sex and Gender in Research

Cell line source(s)	SNU-449 ATCC CRL-2234<br>HLE Cellosaurus CVCL_1281<br>Huh-7 Cellosaurus CVCL_0336
Authentication	Authenticated by short tandem repeat profiling as described by Reid, Y., Storts, D., Riss, T. & Minor, L. in Authentication of Human Cell Lines by STR DNA Profiling Analysis. in Assay Guidance Manual (eds. Sittampalam, G. S. et al.) (Eli Lilly & Company and the National Center for AdvancingTranslational Sciences, 2013).
Mycoplasma contamination	Tested negative for Mycoplasma infection, the test was performed using a PCR-based test (ATCC)
Commonly misidentified lines (See ICLAC register)	No commonly misidentified lines was used or found in this work

Animals and other research organisms

Policy information about studies involving animals; ARRIVE guidelines recommended for reporting animal research, and Sex and Gender in Research

Laboratory animals	Fertilized chicken eggs were obtained from Gepro Geflügelzucht AG
Wild animals	n/a
Reporting on sex	No information regarding the sex of chicken embrions was collected.
Field-collected samples	Fertilized chicken eggs were obtained at day 1 of gestation and were maintained at 37°C in a humidified (60%) incubator for nine days. At this time, an artificial air sac was formed and Huh-7 cells were inoculated on CAMs at 1.5×10^6 cells per CAM, on three to six CAMs each. Embryos were maintained at 37 °C for 4 days after which tumors at the site of inoculation were excised using surgical

	forceps.
Ethics oversight	According to Swiss regulation for animal experimentation, no ethical approval is required for chicken embyos up to 14 days post fertilization.

Note that full information on the approval of the study protocol must also be provided in the manuscript.

Flow Cytometry

Plots

Confirm that:

- [x] The axis labels state the marker and fluorochrome used (e.g. CD4-FITC).
- [x] The axis scales are clearly visible. Include numbers along axes only for bottom left plot of group (a 'group' is an analysis of identical markers).
- [x] All plots are contour plots with outliers or pseudocolor plots.
- [x] A numerical value for number of cells or percentage (with statistics) is provided.

Methodology

Sample preparation	Cells were harvested after the incubation period and washed twice by centrifugation (1,200 g, 5 min) in cold phosphate-buffered saline (DPBS). After washing, cells were resuspended in 0.1 mL AnnV binding buffer 1X containing fluorochrome-conjugated AnnV and PI (PI to a final concentration of 1 ug/mL) and incubated in darkness at room temperature for 15 min. Following immediately, cells were analyzed by flow cytometry, measuring the fluorescence emission at 530 nm and >575 nm.
Instrument	Cells were analyzed by flow cytometry using the BD FACS Canto II analyzer (BD Biosciences, USA https://www.bdbiosciences.com/en-us/instruments/research-instruments/research-cell-analyzers/facscanto-ii)
Software	Data were collected using the BD FACSDiva™ software. Data were analyzed by FlowJo software version 10.5.3 (https://www.flowjo.com).
Cell population abundance	Cell populations were only analyzed but not sorted, therefore no post-sort analysis was required.
Gating strategy	Cells were gated using the SSC-A and FSC-A axis. Single cells were gated using the FSC-H and the FSC-A axis. Live and apoptotic cells were gated using the Annexin V and the PI axis. Live cells were defined as AnnV(-), PI(-); necrotic cells were defined as AnnV(-),PI(+); apoptotic cells were defined as AnnV(+); Specifically early apoptotic cells were defined as AnnV(+),PI(-) and late apoptotic cells as AnnV(+),PI(+).

- [x] Tick this box to confirm that a figure exemplifying the gating strategy is provided in the Supplementary Information.
