# Supplementary material for: Discovery of synthetic lethal interactions from large-scale pan-cancer perturbation screens
Source: Nat Commun. 2022 Dec 14;13:7748. doi: 10.1038/s41467-022-35378-z (PMC9751287; doi:10.1038/s41467-022-35378-z)
Supplement: Supplementary file 4 — Description of Additional Supplementary Files [file 41467_2022_35378_MOESM4_ESM.docx]

**Description of Additional Supplementary Files**

File Name: Supplementary Data 1

Description: Significant SL pairs identified by SLIdR in the pan-cancer and cancer type-specific analyses of DRIVE data.

File Name: Supplementary Data 2

Description: Literature evidence for established and potential SL pairs predicted by SLIdR in pan-cancer and cancer type-specific analyses.

File Name: Supplementary Data 3

Description: Validated pan-cancer and cancer type-specific SL predictions on the PRISM drug-response screen.

File Name: Supplementary Data 4

Description: (i) Significant overlap of predictions with experimentally identified SL pairs. (ii) Consolidated list of experimentally identified SL interactions across 10 combinatorial CRISPR screens. (iii) 1,301 unique experimentally identified SL interactions across 8 combinatorial CRISPR screens.

File Name: Supplementary Data 5

Description: Robust pan-cancer and cancer type-specific SL pairs predicted by SLIdR across DepMap CRISPR and Project DRIVE screens.

File Name: Supplementary Data 6

Description: Key resource table. Reagents used in the functional validation of SL interactions.
